# The effect of a community based health promotion intervention to change gender norms among women in a rural community in Sri Lanka

**DOI:** 10.1186/s12889-018-5914-7

**Published:** 2018-08-06

**Authors:** T. Herath, D. Guruge, M. Fernando, S. Jayarathna, L. Senarathna

**Affiliations:** 1 Department of Primary Health Care, Faculty of Health-Care Sciences, Eastern University, Sri Lanka, No. 50, New Kalmunai Road, Batticaloa, Sri Lanka; 2 Department of Health Promotion, Faculty of Applied Sciences, Rajarata University of Sri Lanka, Mihintale, Sri Lanka; 3 National Child Protection Authority, Colombo, Sri Lanka

**Keywords:** Community based, Gender roles, Gender norms, Health promotion, Violence against women

## Abstract

**Background:**

Gender norms practiced by respective societies increase the risk of violence by men against women. To date, there is a dearth of research evidence on changing gender norms through health promotion approaches around the globe, including in Sri Lanka. This study provides an evaluation of effectiveness of a health promotion intervention in changing the acceptance of gender norms among women.

**Methods:**

A quasi-experimental study was conducted in two rural villages in Anuradhapura district in Sri Lanka including women who have a child under five years of age. One village was allocated to receive an intervention developed based on a health promotion approach and the other village was a control. A community based mechanism to question selected gender norms among women was developed as the intervention. The pre- and post-intervention assessments of the level of acceptance of gender norms were done using an interviewer administered questionnaire and by using focus group discussions.

**Results:**

Following the intervention, acceptances of prominent gender norms were changed significantly among the women receiving the intervention method. The control group showed no changes towards the acceptance of gender norms during this period. Women in the intervention group had higher levels of self-reported positive behavior changes and greater understanding of gender concepts compared to the control group.

**Conclusion:**

The acceptance of gender norms among women in rural villages in Sri Lanka can be changed by a community based intervention targeting gender norms.

## Background

Gender Based Violence (GBV) means any act of violence occurring based on gender that results in, or is likely to result in physical, sexual, psychological or economic harm or suffering for women, including threats or such acts, coercion or arbitrary deprivations of liberty, whether occurring in public or private life [1]. Although, this definition is applicable to both men and women, the phenomenon of GBV mostly effects women [2]. This paper, therefore addresses the issue of violence against women subjected to due to gender by men, known as GBV. The most pervasive form of gender violence is abuse of women by intimate male partners, and this is known as intimate partner violence or domestic violence for this study.

GBV is a major public health issue around the world irrespective of income levels [2, 3]. Women are vulnerable to different types of physical, sexual and psychological violence at different stages of their lives within the family, community and society [4].Globally, 10–60% of women who have ever been married or partnered have experienced at least one incident of physical violence from a current or former intimate partner [5]. In a Sri Lankan study, women attending antenatal and gynecological clinics of a hospital in rural Anuradhapura were screened for domestic violence in 2002, it was found that 40% of them had experienced some form of GBV during their life [6]. Similarly, a survey found 40.7% of women who attended an outpatient department of the teaching hospital, Ragama, in a semi-urban area in the suburbs of Colombo were abused by their male partners [6]. As reported by World Health Organization (WHO), in Sri Lanka GBV ranges from sexual harassment in public places including public transport to acts of violence within the privacy of the home or work place [7].

Gender inequality and discrimination are defined as root causes of violence against women [8]. Evidence suggests that gender inequalities also increase the risk of violence by men against women and inhibit the ability of those affected to seek protection. Further studies have shown that violence against women and girls is related to social norms that prescribe male and female roles in society and condone abuse. Addressing of these gender norms is an important investment to combat the growing issue of violence against women [7–13].

Changing gender norms has demonstrated positive results in combating the issue globally according to the literature [10–19]. Most of these studies were aimed at young couples, boys and men. However, the methods of changing gender norms were limited to interventions such as educational programs, skill development of healthy relationships and public information campaigns. Commonly, these interventions were centralized around individuals. But there is evidence from previous studies that it is important to have coordinated actions at the community level for an effective primary prevention [20].

Many researchers have discussed GBV as a learned social behavior for both men and women [20]. This intergenerational cycle of violence persists because of the traditional social attitudes about women, men and violence. Social traditions have placed women as a housekeeper and a child bearer. Tragedy doesn’t end there, as for women deviation from such roles will bring violence as a punishment [21]. According to previous research, males are perceived as macho beings and aggression as an important macho trait [22]. Further, it is explained that, in South Asian societies, women consider themselves ineffective without males and males should dominate the family. And research has pointed out that husbands try to control wives through violence until they surrender [23, 24].

At present, there is no systematic mechanism for data collection in relation to the prevalence, causes and consequences of GBV in Sri Lanka; however, Sri Lanka has signed all important global declarations and created national policies on prevention of GBV. The Domestic Violence Act of Sri Lanka was passed in 2005, primarily as a response to domestic violence incidents. Also, there is a paucity of research evidence on the most common gender norms that trigger violence in Sri Lanka. However, in patriarchal Sri Lanka, motherhood and marriage are still social norms for Sri Lankan women [25]. And it represents the gender norms and perceptions instilled in Sri Lankan society, which perpetrates not only overt violence but also norms that promote men’s extreme control over many aspects of women’s lives. Gender norms related to motherhood such as caring and nurturing children and preparing food for the family are typical in Sri Lanka [26]. Hence, even minor deviations would trigger violent behavior by males. Existence of masculine attributes associated with money, problem solving, consuming alcohol and tobacco and engaging in household chores bring more value to male control over women [25].

In the context of Sri Lanka, there are preventive measures such as distributing information packages on GBV prevention for newly married couples and for migrant workers particularly travelling to the Middle East countries. In addition, improving the capacity of primary health care workers for detection and support was also implemented by the health sector. But the effectiveness of these measures on reducing GBV was not established due to the lack of scientific evidence. According to a study, the existing literature in the Sri Lankan context is limited to either prevalence studies or literature reviews on domestic and gender violence [27]. But most of the published global and local studies have recommended the need for community based interventions that ensure sustainable outcomes instead of only improving knowledge and attitudes [7, 27]. A key finding of these studies is that targeting women and girls is a sound investment. Women and their families are the main agents of perpetuating gender stereotyping to the next generation, and these agents determine the continuation and prevalence of the existing unhealthy gender norms that contribute to trigger violence when there is a transgression contrary to the norm [28]. In the context of Sri Lanka, women with children under five years are relatively easy to access as they attend field weighing posts conducted by public health field staff under the growth monitoring program of children.

The health promotion approach has been widely recognized as a process of enabling individuals and communities to increase control over their health and improve health status [29]. Health promotion works through concrete and effective community actions to set priorities, make decisions, plan strategies and implement them to achieve better health [30]. The sustainability of such an approach is largely due to increased community ownership.

Hence, it will be worthwhile to check whether, or how, women will take over this approach in preventing GBV. Even though the women are the most common victim of GBV, it remains unclear how this vulnerable group can be strengthened to prevent GBV despite the search for an effective preventive method. This paper presents a study conducted to assess the effectiveness of a community based health promotion intervention for changing women’s acceptance of gender norms in rural villages in Sri Lanka.

The intervention was considered a health promotion intervention as it has a design based on the principles of health promotion. Thus, this paper presents an arena to discuss results of such an intervention.

## Methods

### Setting

This quasi-experimental study was conducted in the Anuradhapura district of Sri Lanka. Anuradhapura district is situated approximately 200 km north of Colombo and has a population of more than 905,000 [31]. It mainly consists of rural areas and their main livelihood is agriculture. The mean income of the monthly income receiver’s income was Sri Lankan Rupees (LKR) (01 USD = 155 LKR) 20,900 in 2012. The median number of years of completed education was 10.1 out of the standard 11 years for ever married women between 15 and 49 years in Anuradhapura in 2013 [32].Two medical officer of health (MOH) areas were selected; one as an intervention and the other as a control community. This is the basic unit of the public health care system of the country and it further breaks into public health midwife (PHM) areas for providing primary care. There are five to ten field weighing posts conducted by PHM to routinely monitor the growth of children who are under five years of age and mothers are requested to bring in their children once a month in both the intervention and control communities. The attendance for these field weighing posts by mothers is high and, thus, accessing this community is comparatively more feasible than any other female community. The selection of intervention and control areas was done by considering socioeconomic backgrounds to reduce differences in basic characteristics. The total population in the intervention and control areas was 49,327 and 42,421 in 2016 [31]. The populations in both intervention and control areas are involved in agricultural work and belong to a rural sector of Sri Lanka. These two groups were selected with a distance of approximately 35 km to minimize contamination of the intervention.

Medawachchiya and Kahatagasdigiliya MOH areas were selected for this study. The intervention and control areas were selected from these MOH areas, respectively. Initially, a PHM area was selected from each MOH area using random number tables and then a field weighing post from each PHM area was also selected using the same method. Siyambalagaswewa, the intervention community is situated in the Poonewa PHM area in Medawachchiya MOH area, and Rotawewa, the control community, is situated in the Samadigama PHM area in Kahatagasdigiliya MOH area.

### Participants

The study group was selected from women with at least one child under five years of age and that attended the selected field weighing posts. Women who had participated in gender related interventions or programs within the last month were excluded from the study. Women were advised to acknowledge if they participated in such interventions before this. This was verified by information obtained through the key informants of the community such as PHM and Grama Niladhari (head of the community). This study did not provide any incentive for the participants. All participated voluntarily.

### Theoretical background of the intervention

The key principles of the health promotion approach [33] and its steps were used as the framework for intervention development. According to these principles, it was necessary to align the intervention as a process of group activities despite the use of individual activities, and identification of the underlying community determinants (UCD) was essential. Empowerment of the women involved was the key principle, while women were encouraged to measure the intervention process and intended changes at individual and community levels. Women will be empowered by identifying UCD and planning and implementing activities to address them.

There are six basic steps in a typical health promotion process. There needs to be an interconnected set of activities by the facilitator to achieve these steps as a process. First, the health promotion facilitator should develop a readiness and motivation among the community towards the targeted change. Second, there should be a mutual agreement among the facilitator and the community about the timely changes of the intervention. Third, UCD needs to be identified by the community. Fourth, the selection of feasible UCD that can be changed needs to be done within the community. Fifth, development of the activities to be conducted within the target group. Sixth, the group must decide suitable indicators to detect the timely changes. Then, the facilitator can monitor the changing process.

### Intervention development

**Fig. 1:**
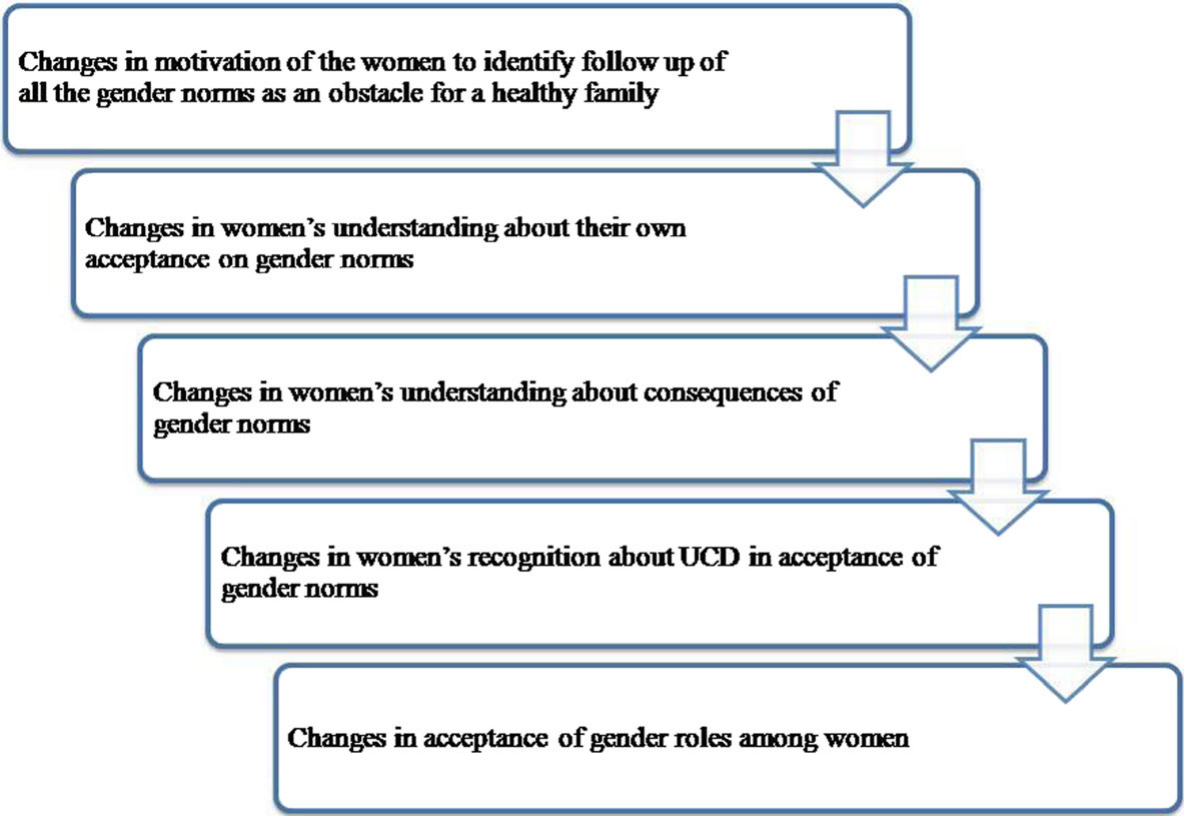
Logical framework of the study. This LFW includes subsequent expected outcomes according to the specific objectives and it was used to facilitate the intervention process towards the intended objectives of the study. Interventional activities were delivered in the sequence of the LFW

The main objective of this study was to assess the effectiveness of a community based intervention that was targeted to change the acceptance of gender norms among women. Specific objectives of the study were to describe the current level of acceptance of gender norms among women, to implement a plan to question the selected gender norms among women and to evaluate the effects of the intervention in terms of changes in the acceptance of gender norms among women. Based on these goals and objectives a logical frame work (LFW) was developed (Fig. 1). This LFW included subsequent expected outcomes according to the specific objectives. Sri Lankan literature on health promotion [33] was used as a reference to obtain a model for the LFW. The existing model was adapted and moderated by the principal investigator (PI) considering specific objectives of the study and approved by the co-authors [2] and experts in the health promotion and GBV fields.

### Selection of six gender norms

Twenty gender norms were identified at the initial expert group meeting and by literature search. The selection of six norms was done in two separate steps. First, marks were allocated on a scale of 1–10 under two criteria by the same experts in their second meeting. These two criteria were potential capacity to trigger violent acts against women in Sri Lankan society and potential health effects associated with each norm. The six norms that received the highest marks were selected to be included in the interviewer administrated questionnaire (IAQ). The same six norms were selected for the intervention. These six norms were related to 1) household chores: women are more responsible for nurturing and caring for children than men, women must perform more household activities than men, women should prepare meals for their families, and 2) socially accepted attributes: women are more frugal than men, behaving violently to a problem is a masculine way as are consuming alcohol and tobacco.

### Intervention structure and delivery

The PI visited the field weighing posts during the survey. Women were informed about the intervention at the very first meeting and were educated about possible health improvements in their families. Then, they were invited to participate in group meetings. Further, they were informed to create community groups in order to deliver the intervention effectively. They were advised to form small groups as they wish and advised to report a date and a time to the PI. So, this group intervention primarily focused on women who showed enthusiasm for the intervention structure at the initial contact (*n* = 20) and the intervention was planned to be extended to other women (*n* = 22) in the community by this active group (total *n* = 42). The extension of the intervention to women who did not participate in the group meetings was used by the intervention as part of the routine schedule of the field weighing post through the PHM. A large group discussion was planned specifically for this group and the women from the active group were motivated to present their experiences and timely progress to the non-participants.

**Fig. 2:**
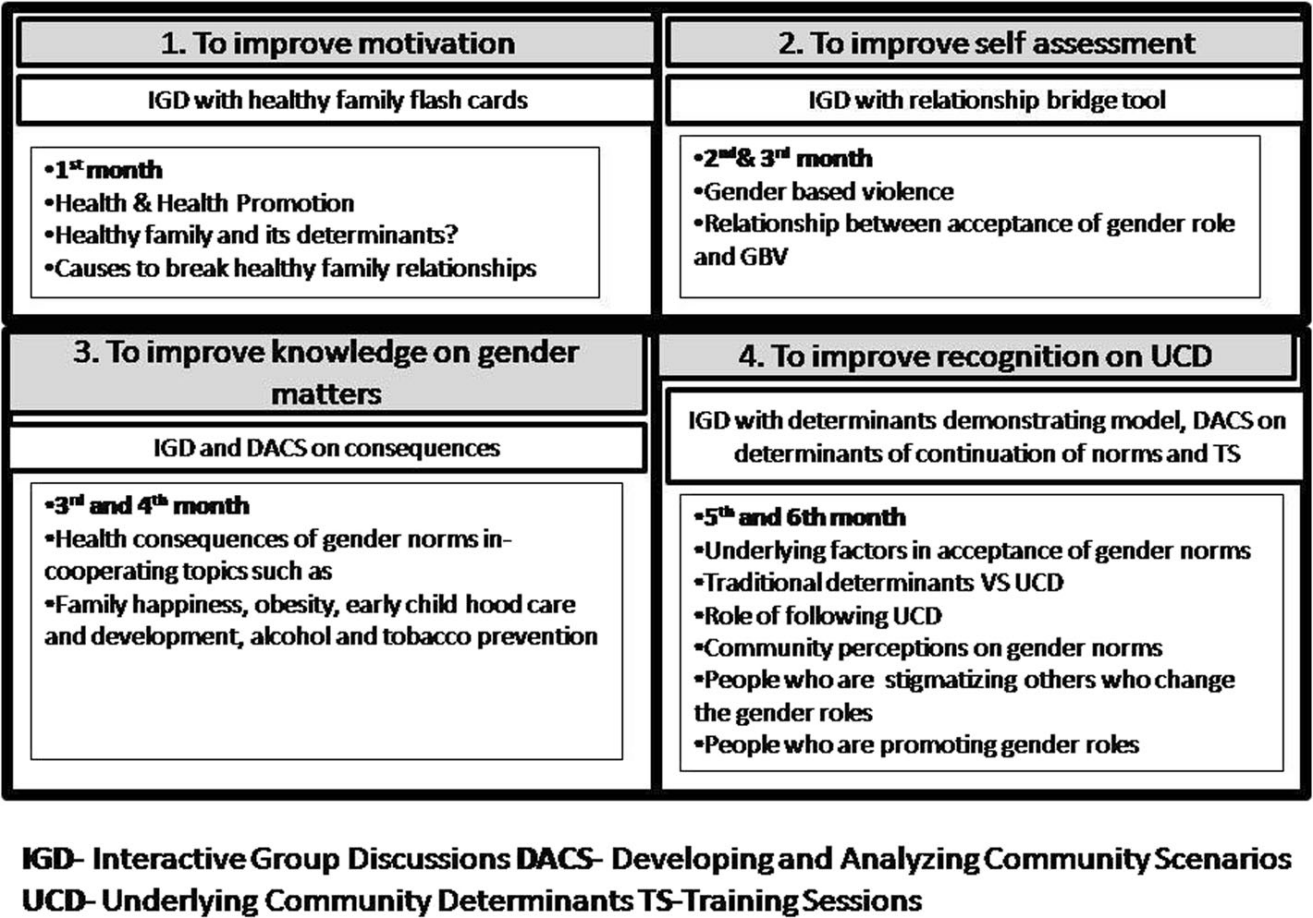
Intervention activities and content with tools used under each step of the LFW (Lesson Plan). Lesson plan demonstrates the type of interventional activity conducted to achieve each step of the LFW with tools. Content of each interventional activity and the specific months in which the discussions carried out have presented under each step of the LFW

The six month period of intervention was designed to build a mechanism within the community to question gender norms, particularly the six selected norms. The LFW was used to facilitate the intervention process towards the intended objectives of the study. Intervention activities were delivered in the sequence of the LFW. These interventional activities included interactive group discussions, developing and analyzing community scenarios on the consequences and determinants of the continuation of gender norms and training sessions on skill development to enable women to question gender norms. The PI carried out interventional activities with four groups of women (*n* = 5 per group) whom were selected from the participants at the field weighing post. The PI is a BSc (Special) Health Promotion degree trainee and she had extensive, two years, of field work experience for community health promotion work. The PI’s past experience was used to develop intervention activities and the activities were supervised by the co-authors [2]. These groups actively participated in at least three meetings per month on average in order to achieve the intervention. Out of these three meetings, two meetings were conducted in either of the group member’s home (eight visits per month by the PI for four groups) and other meeting was conducted at the weighing post day with the involvement of all 42 women. For each activity there was a separate lesson plan (Fig. 2).

### Detailed information about the intervention activities, content and tools under each step of the LFW (lesson plan) (Fig. 2)

To improve motivation (LFW Step 1):

1. The topic ‘Health’ was discussed with women as a ‘state of complete physical, mental and social well-being and not merely the absence of disease and infirmity’. The aim was to broaden their view about health and persuade them to think about their own health as well as to motivate the need to improve their own health.
2. The topic ‘Health Promotion’ was introduced as a process that will provide them a capacity to make decisions, to plan and to be active in their own health improvement.
3. A healthy family was introduced to them as a happy family that has a healthy physical environment, healthy children, healthy body, healthy expenditures and, most importantly, healthy relationships. These were considered as determinants of a healthy family.
4. Causes to break healthy family relationships were a leading topic discussed. Gender norms were proposed as one of the factors and women were facilitated to assess their own family status in terms of gender norms, family happiness and vulnerability to loss of family happiness. Moreover, women were led to compare potential harms embedded in gender norms with the potential benefits of changing.

To improve self-assessment (LFW step 2):

1. The topic “GBV” was used as the initiation to improve women’s understanding about their own acceptance of gender norms. First, women were facilitated to reflect their own understanding of GBV and to produce examples of GBV. Finally, the topic of GBV was described as any act that results in, or is likely to result in, physical, sexual, psychological or economic harm or suffering based on the gender of a person.
2. At the next step, gender roles were highlighted as the root cause for GBV by showing a breakdown of causes for a real life scenario about an act of GBV. The relationship between acceptance of GBV and gender roles was discussed as the final step.

**Fig. 3:**
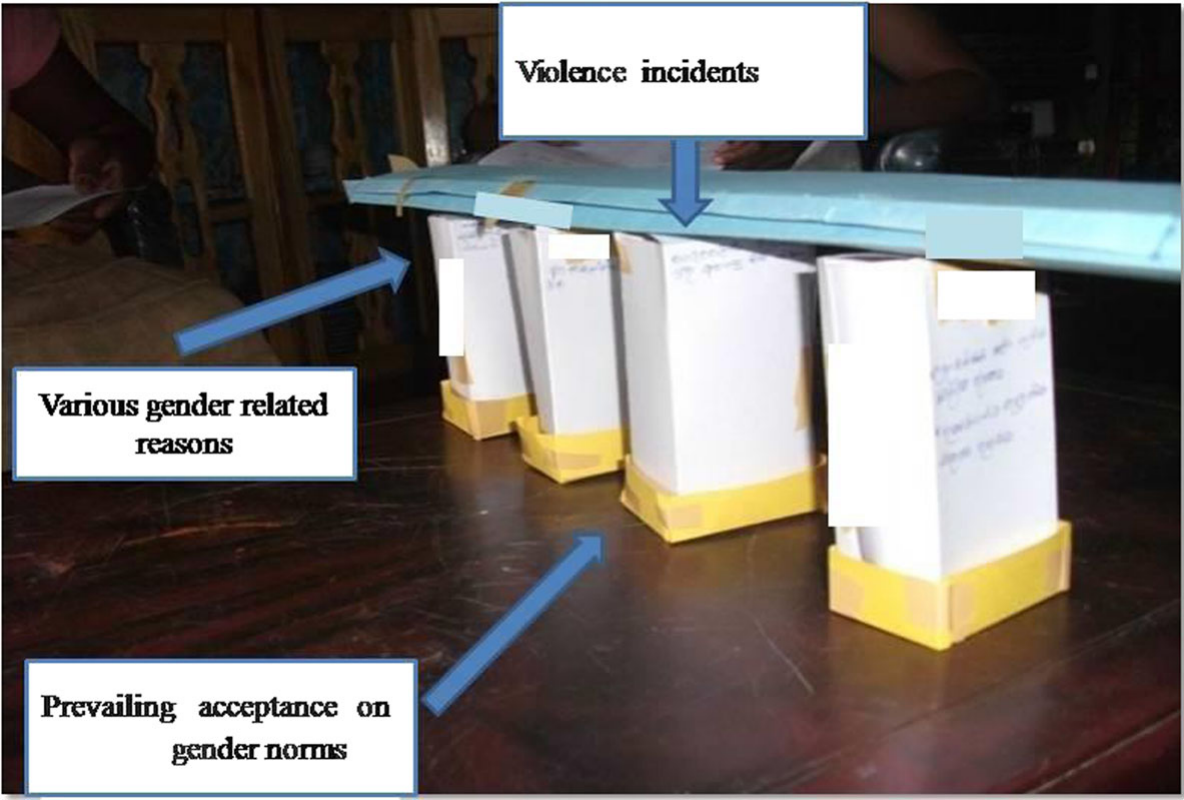
Relationship bridge. This is an innovative intervention tool that was developed by the PI to facilitate the process of the discussion to achieve the second step of the LFW. This tool was used to show the relationship between women’s acceptance on gender norms and violent incidents in the family or community. The tool consists of three components: a top layer to showcase potential violent incidents, middle pillars to represent various gender related reasons and a ground layer that works as a foundation for the bridge to present prevailing acceptance on gender norms among women

These discussions were facilitated with innovative intervention tools that were developed by the PI to facilitate the process of the discussion. One of the tools was ‘the relationship bridge’ (Fig. 3). This tool was used to show the relationship between women’s acceptance on gender norms and violent incidents in the family or community. The tool consists of three components: a top layer to showcase potential violent incidents, middle pillars to represent various gender related reasons and a ground layer that works as a foundation for the bridge to present prevailing acceptance on gender norms among women. Women were first informed to measure the level of acceptance on gender norms among their peers. After they analyzed it, this tool was used to connect violent incidents identified against women with the values they obtained for each norm.

To improve knowledge on gender matters (LFW step 3):

Discussion about health consequences of gender norms were done at this stage. Consequences such as low male contribution to child growth and development, communication barriers between children and male members of the family, unwanted expenditures like alcohol and tobacco, increased risk behaviors among men, high suicide rates among men, higher rate of sexually transmitted diseases among men, reduction of family happiness, increasing numbers of times getting angry and many other issues were discussed. Real life scenarios were presented, which led women to identify consequences and perceived severity of these gender norms. This was done by cooperating with the topics family happiness, early childhood care and development, alcohol prevention and tobacco prevention. This incorporation of health perspectives helped participants to achieve each step of the LFW as all the topics were closely linked to their overall well-being.

**Fig. 4:**
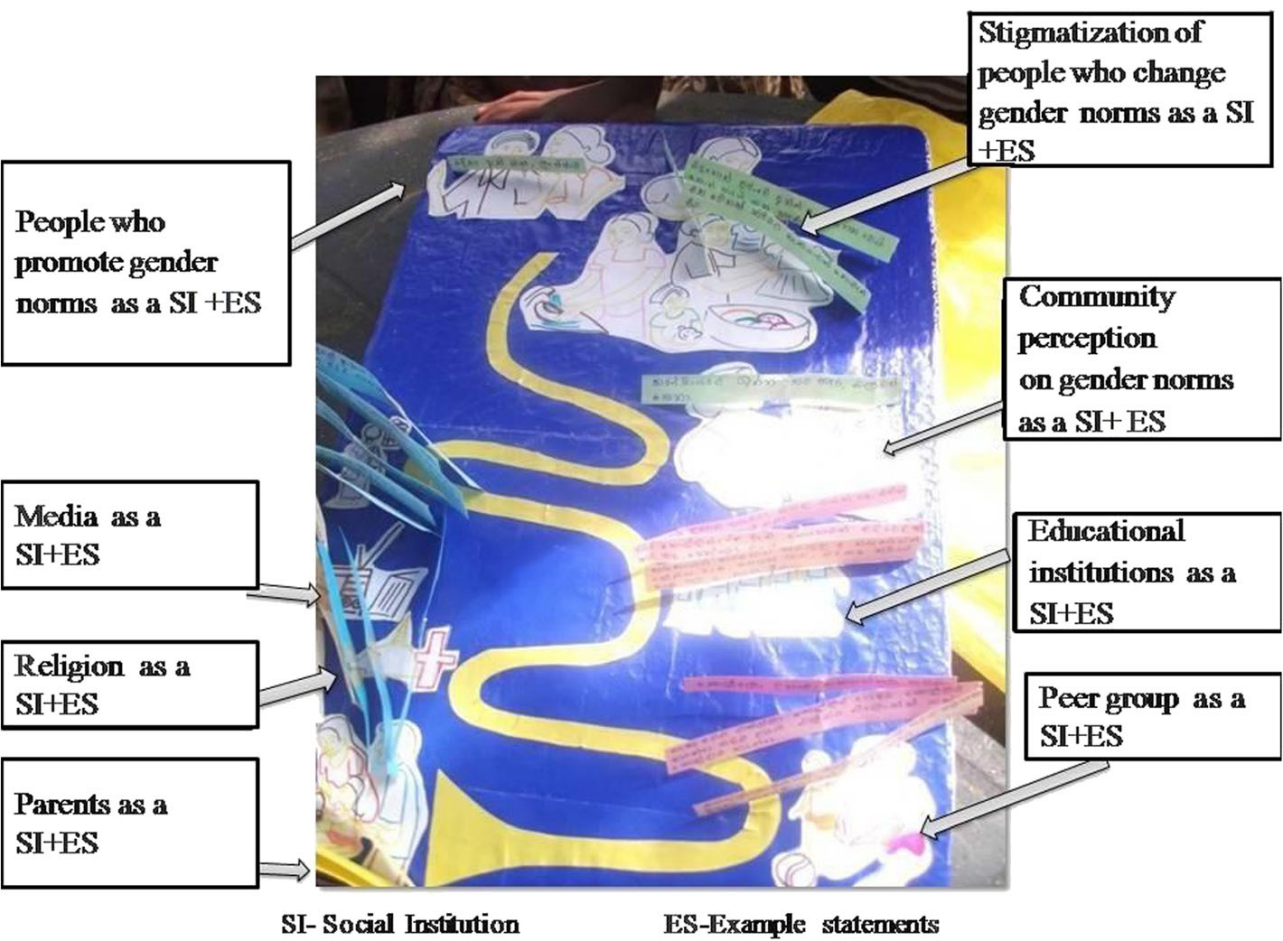
Determinants demonstrating model. This is an innovative intervention tool that was developed by the PI to facilitate the process of the discussion to achieve the last step of the LFW. This tool consists of selected social institutions and sample statements about how gender norms are created and how gender norms are influenced by these selected social institutions

To improve the recognition of UCD (LFW step 4):

As the final but most crucial step, underlying factors in acceptance of gender norms were discussed. Discussions were first started by discussing the traditional underlying factors from the previous literature such as the role of social institutes (family, peer group, education, economy, religion and media) on molding one’s perceptions on gender roles. Role of UCD was discussed among women as the last step. For this step a tool called ‘the determinants demonstrating model’ (Fig. 4) was used. This tool demonstrated how gender norms are created and the influence of different social institutions. Women were engaged with the tool to reveal real statements of selected social institutions on gender norms. These statements were used in discussion to broaden women’s understanding of UCD. Women were facilitated to identify people who promote gender norms, stigmatization of people who deviate from gender norms and embarrassment and fear in changing gender roles as overt UCD facilitators. The women developed real life scenarios based on these overt UCD, which facilitated the development of a response scenario that was practiced throughout training sessions.

Simultaneously with the intervention activities, women were motivated to question the gender norms in their life settings such as family conversations and common community gatherings such as in shops, bathing places and community functions. They had been empowered to measure changes in acceptance of gender norms among themselves and their own questioning process by using recording charts that were developed by the groups themselves. The PI did periodic monitoring to detect timely progress and impediments. Identified details were used to scale up the intervention further and to plan necessary motivation. Sustainability of the intervention was ensured by forming a secondary active group from rest of the population and training them to report timely outcomes to the first active group. Active groups discussed their progress, obstacles and way forward.

### Study instruments

The questionnaire was pretested by administering it to a sample of women with similar characteristics, such as age range, educational level and occupation, to the original sample to detect any problems related to unclear wording or understandability of the questions and to check the average response time. This pre-tested IAQ was used for data collection. The questionnaire was developed to suit the Sri Lankan context by incorporating local experts’ views on gender norms and by referring to previous literature to detect prominent gender norms. This study assessed the acceptance of gender norms in terms of six selected gender norms. For each norm, participants could respond on a five point Likert scale ranging from *totally agree, agree, can’t say, disagree* and *totally disagree.* Focus group discussions (FGD) were conducted to understand self-perceptions on behavior changes following the intervention as an outcome measure of the change in acceptance according to standard procedures.

### Data collection

The research team met both intervention and control group participants at their homes for both pre- and post-evaluations. During these evaluations, demographic and socioeconomic data (age, level of education and employment status of both wife and husband) of the participating women and their level of acceptance of the six selected gender norms were collected. Pre- and post-evaluations were conducted prior to commencing the intervention and six months after the intervention.

Focus group discussions in the intervention group were conducted with two groups of women selected using purposive sampling. As the basic intervention was delivered to the active group, it was decided to select this group for the FGD. Women who showed enthusiasm for the group intervention at the initial contact and those who were the primary audience of the intervention (*n* = 20) were invited through a notice. Those who were willing to participate were randomly divided into two groups (*n* = 9). All participants were housewives and their age ranged from 23 to 42 years. For the FGD, a predefined date and location were provided to them and the research team obtained data on behaviors related to selected gender roles and behavioral changes that occurred due to the intervention among them and their family (for the post-evaluation only). The FGD guide consisted of two sections. The first section included questions intended to provide information about their understanding of gender concepts and its relationship with GBV. The second section was on changes that occurred after the intervention. Sub-questions were based on changes in relation to daily routine, changes in terms of support from male members, changes of behaviors among women and attempts to change gender roles. The women were informed to discuss their own attitudes in relation to each question. The PI moderated the FGD and allowed free discussions within the group. All the discussions were held in Sinhala language. They lasted on average 60 min and were recorded after getting approval from the participants. Saturation method was used to determine the number of groups involved for the data collection for the qualitative study. We observed that the same information was being repeating during the last leg of the second discussion and decided that we had achieved data saturation on this topic for this group. A similar method was followed to conduct focus group discussions in the control group.

### Data analysis

The characteristics of the study population were summarized and described by using descriptive statics and the comparison of the characteristics of intervention and control groups was done using Chi square test. For analysis, strongly disagree and disagree were combined and renamed as disagree and strongly agree and agree were combined and renamed as agree. Similarly, Chi square test was used to detect statistically significant differences among pre- and post-levels of acceptance of gender roles in the intervention and control groups, namely 1) pre-level of acceptance of gender roles in intervention group and pre-level of acceptance of gender roles in control group, 2) pre-level of acceptance of gender roles in intervention group and post-level of acceptance of gender roles in intervention group, 3) post-level of acceptance of gender roles in intervention group and post-level of acceptance of gender roles in control group and 4) pre-level of acceptance of gender roles in control group and post-level of acceptance of gender roles in control group. Statistical Package of Social Sciences (SPSS) 20 statistical software was used to analyze the quantitative data.

All interviews were recorded and transcribed. The transcribed interviews were then read, coded and analyzed to extract themes. Thematic analysis was conducted concurrent to fieldwork and both data collection and analysis followed an inductive approach, whereby investigation was refined and revised to fit the evolving data and findings. Furthermore, the principles of grounded theory were followed during the coding and the process of generating data. The PI of the research conducted independent coding, analysis and developed an overall interpretation of the data.

### Ethical considerations

Ethical clearance was obtained from the Ethical Review Board of the Faculty of Medicine, Rajarata University of Sri Lanka. Informed written consent for participation was acquired prior to recruitment to the study. Specific measures were taken during the study to improve the confidentiality of data. Due to the nature of the interventions, collecting personal information from participants was necessary. However, precautions were taken to maintain anonymity by assigning a unique serial number to each participant when data was entered in to a database and prior to the analysis. No personal identifications are revealed during presentations. Data was stored safely in password protected databases and spaces with limited access.

For safety reasons, when obtaining community support for this research it was considered important to frame the study in general terms such as a study on women’s health or life experiences rather than mention violence or abuse directly. In order to protect the respondents, the study was introduced as a family well-being improving project to the community and to the family, with only the respondents (women) being told the true nature of the project. Women were encouraged to reflect to their own status and connect with peer circles to discuss changes in gender norms to reduce possible distress caused to the participants by the research. Women were not encouraged to address power relationships directly and all the intervention activities were linked to health and family well-being development. All activities by the empowered women emerged through peer group discussions and women were empowered to involve their families, and each month progress was measured.

Female data collectors with training in community work were recruited and trained in counseling and methods for working with abused women. Data collection was conducted in the respondent’s home at a convenient time, where it was possible to ensure complete privacy. The local field weighing post was used as an alternative venue for the interview when necessary. The respondents were offered contact and referral information for centers providing services for abused women in instances where it was safe to receive and keep such information.

A method of providing ongoing support and referral to women facing GBV was discussed at periodic progress monitoring sessions with all the authors. Follow up sessions were done in cases that identified serious domestic violence that affected women and/or children and individual monitoring sessions were conducted concerning their family level interventions and about its progress. The same intervention program was offered to the control community at the end of the study.

## Results

### Characteristics of the study participants

There were 42 and 30 women from the field weighing posts selected as the intervention and control groups, respectively. The response rate within the study population was 100% and all were included into the study. Participants were invited at the field weighing post by both the PHM and PI. Also, a personnel notice was sent to their homes prior to the data collection. Both measures ensured the response rate.

The highest proportion of women belonged to the 27 and above age group in the intervention and control groups. More than 72% of women in each group were housewives. In both groups, more than 50% of women had completed school education up to the ordinary level. In both groups, the husband of the household was occupied in a permanent job, particularly in the security forces. According to the Chi square test, there were no statistically significant differences between the intervention and control groups in relation to women’s age groups, women’s and men’s occupation and women’s educational levels (Table 1).

**Table 1:** Descriptive Characteristics of the study population

Characteristics	Intervention group (%)	Control group (%)	χ2	*P* value*
Age groups (Yrs)				
19–22	4 (9.5)	3 (10.0)	2.501	0.29
23–26	11 (26.3)	13 (43.3)		
27 and above	27 (64.2)	14 (46.7)		
Occupations of the women				
Housewife	34 (81.0)	22 (73.3)	1.685	0.43
Farmer	8 (19.0)	7 (23.3)		
Other	0 (0)	1 (3.3)		
Education levels of the women				
Up to grade 8	8 (19.0)	5 (16.7)	1.410	0.40
Pass GCE Ordinary Level	21 (50.0)	19 (63.3)		
Pass GCE Advanced Level	13 (31.1)	6 (20.0)		
Occupations of the husbands				
Security forces/police	31 (73.8)	16 (53.3)	5.120	0.08
Farmers/carpenters	8 (19.0)	13 (43.3)		
Government office workers/teachers	3 (7.2)	1 (3.4)		
Total	42 (100.0)	30 (100.0)		

*Significance level ≤ 0.05

### Acceptance of gender norms among women in pre-evaluation

The majority of the participants in both groups had the same level of acceptance of the selected gender norms. There was no significant differences in women’s acceptance of gender roles between the intervention and control areas. However, most of the participants in both intervention and control groups showed their disagreement on the gender norm of alcohol and tobacco use by men, even though the majority of them showed an agreement towards other gender norms (Table 2).

**Table 2:** Acceptance of gender norms among women in pre-evaluation

Norm	Intervention pre-evaluation (%)	Control pre-evaluation (%)	*P* value*
Women are more responsible for nurturing and caring for children than men			
Agree	38 (90.4)	28 (93.3)	0.67
Disagree	4 (9.6)	2 (6.6)	
Women are more frugal than men			
Agree	42 (100.0)	29 (96.6)	0.23
Disagree	0 (0.0)	1 (3.3)	
Behaving violently to a problem is a masculine way			
Agree	29 (69.0)	24 (80.0)	0.29
Disagree	13 (30.9)	6 (20.0)	
Women must know more household activities than men			
Agree	40 (95.2)	26 (86.6)	0.19
Disagree	2 (4.8)	4 (13.4)	
Women should prepare meals for the family			
Agree	25 (59.5)	20 (66.6)	0.54
Disagree	17 (33.5)	10 (33.3)	
Consuming alcohol and tobacco is a masculine way			
Agree	5 (11.9)	11 (36.6)	0.06
Disagree	37 (88.0)	19 (63.4)	
Total	42 (100.0)	30 (100.0)	

*Significance level ≤ 0.05

### Changes in acceptance of gender norms among women in post-evaluation for intervention group

Five out of six selected norms showed a significant change in acceptance during the pre- and post-interventional comparison. The acceptance of the norm “Behaving violently to a problem is a masculine way” only slightly changed and that change was not significant (Table 3).

**Table 3:** Changes in acceptance of gender norms among women in intervention group at post-evaluation

Norm	Intervention pre-evaluation (%)	Intervention post-evaluation (%)	*P* value*
Women are more responsible for nurturing and caring for children than men			
Agree	38 (90.4)	25 (59.5)	0.00
Disagree	4 (9.50)	17 (40.50	
Women are more frugal than men			
Agree	42 (100.0)	28 (66.6)	0.00
Disagree	0 (0.0)	14 (33.4)	
Behaving violently to a problem is a masculine way			
Agree	29 (69.0)	25 (59.5)	0.36
Disagree	13 (30.9)	17 (40.4)	
Women must know more household activities than men			
Agree	40 (95.2)	22 (52.3)	0.00
Disagree	2 (4.8)	20 (47.7)	
Women should prepare meals for the family			
Agree	25 (59.5)	10 (23.8)	0.00
Disagree	17 (33.5)	32 (76.2)	
Consuming alcohol and tobacco is a masculine way			
Agree	5 (11.9)	0 (0.0)	0.02
Disagree	37 (88.0)	42 (100.0)	
Total	42 (100.0)	42 (100.0)	

*Significance level ≤ 0.05

### Changes in acceptance of gender norms among women in post-evaluation of control group

According to the data, there were no changes to the acceptance of selected gender norms among women in the control group during the study period. The level of acceptance to all gender norms stayed same except for two (Table 4).

**Table 4:** Changes in acceptance of gender norms among women in control group at post-evaluation

Norm	Control pre-evaluation (%)	Control pre-evaluation (%)	*P* value*
Women are more responsible for nurturing and caring for children than men			
Agree	28 (93.3)	28 (93.3)	1.00
Disagree	2 (6.7)	2 (6.7)	
Women are more frugal than men			
Agree	29 (96.6)	28 (93.3)	0.55
Disagree	1 (3.3)	2 (6.7)	
Behaving violently to a problem is a masculine way			
Agree	24 (80.0)	22 (73.3)	0.54
Disagree	6 (20.0)	8 (26.6)	
Women must know more household activities than men			
Agree	26 (86.6)	26 (86.6)	1.00
Disagree	4 (13.3)	4 (13.3)	
Women should prepare meals for the family			
Agree	20 (66.6)	19 (63.3)	0.79
Disagree	10 (33.3)	11 (36.6)	
Consuming alcohol and tobacco is a masculine way			
Agree	11 (36.6)	12 (40.0)	0.79
Disagree	19 (63.4)	18 (60.0)	
Total	30 (100.0)	30 (100.0)	

*Significance level ≤ 0.05

### Changes in acceptance of gender roles among women in post-evaluation among intervention and control groups

Significant differences were noted between the proportions of women from the intervention and control groups who accepted selected gender norms in the post-evaluation. Such changes were not observed for only one norm (Table 5).

**Table 5:** Changes in acceptance of gender roles among women at post-evaluation in intervention and control groups

Norm	Intervention post-evaluation (%)	Control post-evaluation (%)	*P* value*
Women are more responsible for nurturing and caring for children than men			
Agree	25 (59.5)	28 (93.3)	0.00
Disagree	17 (40.5)	2 (6.7)	
Women are more frugal than men			
Agree	28 (66.6)	28 (93.3)	0.01
Disagree	14 (33.4)	2 (6.7)	
Behaving violently to a problem is a masculine way			
Agree	25 (59.5)	22 (73.3)	0.22
Disagree	17 (40.4)	8 (26.6)	
Women must know more household activities than men			
Agree	22 (52.3)	26 (86.6)	0.00
Disagree	20 (47.7)	4 (13.3)	
Women should prepare meals for the family			
Agree	9 (21.4)	19 (63.3)	0.00
Disagree	33(78.6)	11 (36.6)	
Consuming alcohol and tobacco is a masculine way			
Agree	0 (0.0)	12 (40.0)	0.00
Disagree	42 (100.0)	18 (60.0)	
Total	42 (100.0)	30 (100.0)	

Significance level ≤ 0.05

### Self-reported behavior changes from FGD

Women in the intervention group had higher levels of self-reported positive behavior changes and greater understanding of gender concepts. According to data, they were able to encourage other family members to change gender roles. It was also noted that these women made efforts to share household work with their husbands following the intervention according to their accounts. Positive behavioral changes were noticed from the two norms “Women are more responsible for nurturing and caring for children than men” and “Women must know more household activities than men”, while other norms showed less positive behavioral changes. These changes were revealed during the FGDs and seemed to be common for many women who were in the intervention group. The themes and supporting quotes showed that the women who took part in the intervention were empowered enough to discuss and challenge gender norms with their partners or family.

### Theme 1: Sharing household work with their husbands

#### Intervention group

Before the intervention, the women were unlikely to ask for or gain support for routine household work by their partners. But after the intervention, they requested support from them rather than following the traditional roles of men. According to past Sri Lankan and global studies, reasons for not disclosing abuse and not questioning typical concepts of gender roles included embarrassment and fear of more violence [34, 35]. They were also concerned about family reputation. But, results clearly indicated that the intervention effectively empowered women to change acceptance of gender roles by addressing obstacles identified through past studies.

*“Earlier I had to stay all the time in the kitchen. But now I have managed to divide work between my husband and me. When I am doing children’s work he cuts the vegetables.*” (Woman 1, 30 years old).

#### Control group

These women were unlikely to ask for or gain support for routine household work by their partners. Women in the control group followed the traditional roles of men.

*“In our village women get up early and cook. Then ready children to go school and then sweep the home. Then wash clothes. If they have a cultivation she also wants to look after that. If the male comes home to lunch she serves him the food. After he goes she feeds the children. This is not end; this is same in all the days in the week. Men go for the job, after coming they were getting a bath and watch television or chat with friends. They also look on children, but when mother is there he avoid from it.”* (Woman 4, 31 years old).

### Theme 2: Encouraging children to change gender roles to change gender stereotyping

#### Intervention group

With the progress of the intervention, women changed their acceptance in view of initiations to engage their boy children in activities labeled as girl children’s activities and vice versa. As GBV is a learned behavior when children are exposed to violence between their parents, boys learn violence as a means of achieving control and girls learn to accept violence. In this context, effective inputs to change the root causes of GBV will bring positive influence to break the learning cycle among children and, as the author feels, women are effectively empowered to break this cycle among their children.

“*Earlier our son was not playing in play houses like girls; we did not push him to do either. But after this program I let my son to play with dolls and to cook in clay pots.*” (Woman 2, 35 years old).

#### Control group

Women in the control group perceived that socially determined gender roles should be taught at home before exposing children to society.

*“Jobs of women are cooking, washing, cleaning and looking after the children. Men should go for a job and they should find the income to the family. This truth need to teach at our homes to our children. We can’t go beyond what society determined.”* (Woman 2, 29 years old).

### Theme 3: Teaching the knowledge learned from intervention activities

#### Intervention group

It was revealed that women had engaged in teaching activities inside their families to disseminate what they learned via the intervention. This was used as a strategy to change family member’s prevailing acceptances on gender norms. It was also pointed out by Sri Lankan researchers that women themselves accepted violence as a normative behavior and this was reinforced by family and friends [25]. Having such obstacles, it was evident that the intervention effectively empowered women to change acceptance of gender roles by addressing obstacles in their immediate social environment.

*“Earlier I had to look after the child when I was in the kitchen too…My husband used to watch television or chat with neighbors. I taught him about Early Child hood Care and Development…and ask him to stay with the child and to give stimulations for the child by looking after him.”* (Woman 3, 25 years old).

#### Control group

There were no efforts to change gender roles among their families. They were continuing the gender concepts without questioning and normalization, hesitancy and fear to stigma were the major factors for not intervening to change gender concepts.

“*Everybody has problems in their homes. But we must save our traditions. If we try to change other people in the village will laugh at us and our family will face embarrassment in the village.”* (Woman 1, 27 years old).

### Theme 4: Family conversations to suppress pressure from elders

#### Intervention group

Pressure from elders appeared as an obstacle when the intervention progressed. Elders continually pressured adherence to tradition. Women started family conversations to suppress this pressure irrespective of being silent in front of elders as they practiced earlier. Lack of external support, social stigma and women’s hesitancy to challenge the patriarchal norms were identified as associated factors in tolerating violence by Sri Lankan wives [36]. In this respect, the intervention was effective in empowering women to relieve themselves of a key root cause of GBV.

*“My husband is interested in holding the child…But his parents had discouraged him whenever he held the child to support me. His parents had an attitude such as holding a child to support to his wife is similar to practicing the women’s role. I questioned whether men are prohibited to hold the child. Is that only a women’s thing…”* (Woman 4, 32 years old).

#### Control group

Women in the control group identified elders who promote gender roles as people who guide them in the correct way. Questioning their behavior seemed to be associated with stigma and fear.

“*One day, I was late to prepare the lunch on time as I was engaged in a discussion with mothers of my kid’s pre-school. My husband had to wait till I prepare the lunch and he shouted at me. The mother-in-law told me that I must have done something wrong for my husband to shout at me. How can I tell, I was talking about my child at the preschool. If I didn’t accept the fault, I might be stigmatized by the family.”* (Woman 2, 30 years old).

## Discussion

Our findings indicate that the health promotion intervention developed for this study successfully changed the acceptance of gender norms among women in this community. Prior to the intervention, acceptance on selected gender norms among women was high. But the post evaluation has shown that participants in the intervention group have less acceptance of most of the gender norms (5 out of 6), whereas no similar change was observed in the control group. This finding is consistent with other studies of gender norms changing interventions irrespective of the mode of intervention, whether it is a social, economic or combined [19, 37–41].

This study measured the acceptance of prominent gender norms that were viewed to ‘trigger’ violence against women in Sri Lanka. Perceptions of being a good wife were identified by several past Sri Lankan studies [35, 42], and these provided solid support for the selected norms by explaining masculine attributes in a patriarchal society. According to these, the family expects wives to continue norms related to motherhood and marriage. Further, women also believe adherence to such norms will protect them against violence and at the same time will uphold the reputation of the family. Studies from throughout the world also identified events that represent transgressions of dominant gender norms [4], such as a wife not obeying her husband, talking back, not having food ready on time, failing to care adequately for the children or home, questioning him about money or girlfriends, going somewhere without his permission and refusing him sex. When considering the dominant gender norms that trigger violence, there were more than 70% of Sri Lankan women who totally agreed with both norms “Women are more responsible for nurturing and caring for children than men” and “Women are more frugal than men”. There were more than 74% women who agreed that “Women must know more household activities than men”. This acceptance denotes the traditional division of work among males and females in a Sri Lankan context, and the current study results are comparable with other research evidence in the world on incidents that trigger violence [35, 42]. Partner’s alcohol consumption was identified as a possible risk factor for violence. Behaviors associated with substance abuse are more acceptable in South Asian communities [43]. The majority of women in this study, in contrast, were taught that consuming alcohol or tobacco isn’t an attribute of masculinity.

The gender norm that “Behaving violently to a problem is a masculine way” didn’t change according to the findings. This norm is likely to be the most rooted gender norm among female communities as it is evident in the global studies as well [4]. According to a previous study conducted in Sri Lanka, there is an association between wives’ attitudes toward gender roles and intimate partner violence [42]. The wives were less likely to experience current abuse by husbands if they believed traditional gender norms. The influential nature of the norm may be a reason for not being successful in changing the above norm.

Apart from quantitative measures, we were able to explore changes from a qualitative perspective, which is one of the strengths of the study. Behavioral changes identified from the FGDs would be the strongest indicators of empowering women to put them in a position to be agents of change within the family and community. The claims of getting increased support from their husbands for domestic work and even for child caring, despite the negative remarks from in-laws, also indicated that these women could come out of the prescribed conventional gender role.

As an implication, these types of interventions can be modified by adapting validated ongoing monitoring systems to track changes in the process, in order to measure behavioral changes in a quantitative basis. But in reality there are no validated instruments to track such changes. So, further research is needed on this regard in a Sri Lankan context. Based on these results, further modified interventional research will be needed to change the unchanged norms as well to accelerate the process to change the behaviors regarding other norms.

GBV is a complex phenomenon shaped by antecedents operating at different levels, and gender plays a fundamental role. According to studies, this has important implications for the design of interventions to address GBV [4]. A recent review highlighted that it is easier to increase awareness and modify attitudes than to change violent behavior [16]. Similarly, this study also dealt with perceptions as the major priority while targeting behaviors as an indirect result. Furthermore, according to this review community wide interventions are required to prevent violence and these should address social and cultural factors at the community level. According to several studies, mobilizing communities by targeting both young males and females will be a key in reducing levels of violence [7, 8, 15, 17]. This study also focused on women who have children under five years of age. In Sri Lanka, this group of women are more likely to be enthusiastic of their well-being. Therefore, it is necessary to ensure the adaptability of the current intervention for other women groups in general. Also, many researchers have pointed out that rural sector is more prone to have GBV [16] and, hence, this study was conducted with a rural setup. So, applying the results to an urban setting might require modifications.

Though a number of successful projects have applied community based approaches for changing gender norms, there is limited evidence to date for utilizing a health promotion approach to mobilize communities to use the concept of community empowerment to change behaviors. According to the literature, most violence prevention programs for empowering women and girls in low and middle income countries (LMIC) consist of a series of educational workshops to ensure participatory group training [44]. Furthermore, these interventions were embedded in programs that aimed to improve sexual and reproductive health and livelihood programs, such as microfinance or vocational training. Irrespective of the aim, similar to our study, addressing masculine and feminine attributes was the goal. Moreover, violence prevention programs in LMIC also used approaches such as social communication and community mobilization. And many intervention models function in cooperation with HIV prevention programs. Hence, some studies used integrated approaches to combat gender norms as a step to reduce the prevalence of GBV. As a result, past interventions ranged from social, economic or combined interventions [40].

It was also evident from the literature review that most of the studies reported findings in relation to violence and rarely described changes in gender norms quantitatively, though gender roles were the targeted areas to change. Group training interventions for women and girls were conducted by several researchers and most of the studies were conducted quasi-experimental studies. These interventions consisted of life skills courses [45], empowerment and self-defense programs [46]. All studies demonstrated reduced violence against women after the interventions. In contrast, group training interventions were conducted with women and men through a 50 h training that included addressing gender norms [47]. But, results weren’t consistent for both women and men. Furthermore, there was a reduction in violence by community mobilized interventions for both women and men through educational programs [19, 48] and interventions based on psychological models [49]. According to our literature search, health was included in only one study as a theme [48]. Even though these studies presented results in relation to reduction in violence, the extent to which the gender norms were changed by interventions was not evaluated. In contrast, our study centralized around presenting the extent of changing gender norms via a health promotional intervention. As a secondary step we investigated the behavioral changes in relation to gender norms after the intervention. Therefore, there were considerable differences in between our study and past studies. Notably, our study was not a total educational program but an intervention based on a health promotion approach that was scaled up from knowledge improvement sessions to community planning sessions. Different health topics were used in cooperation to achieve the defined change in relation to each LFW step in our study.

Despite of the results achieved by most of the past studies, they lacked a proper theory and conceptualization or LFWs that could be used to explain the changes gained [14]. By contrast, this study was conducted according to the health promotion principles including a community based strategy involving women, community empowerment, community led mechanisms to maintain a high follow up rate, addressing UCD and incorporation of gender norms to cover all domains to address more than one type of GBV. Moreover according to the LFW, the acceptance of five norms among women were changed due to the change in their acceptance of the norms, starting question their basis and finally starting to question underlying factors of acceptance of gender roles. Development of the LFW made it easier for the PI to facilitate women to achieve the above changes in due time.

This community empowerment concept through a health promotion approach is novel to Sri Lanka. Hence, the findings were not directly comparable with previous studies conducted in Sri Lanka as no study had been done to change the acceptance of gender roles among women in Sri Lanka. This study found five easily changeable gender norms out of six prominent gender norms in Sri Lanka through an intervention that used the health promotion approach. In comparison, this is one of the first quasi-experimental studies to explain the behavior of each single gender norm after facing an intervention while other studies explained the change as a whole.

In addition to providing new data, our study also addressed several limitations identified by past literature related to interventional research on changing gender norms to date. The study included a comparison group, concern on gender norms related to various aspects and types of GBV, initiatives to maintain follow up and theory based designing of the intervention. Though the present study did not measure the impact of the initiatives on violence against women, this study collected information on supportive behaviors to reduce the problem. Furthermore, standardized or validated measures using a locally validated data collection tool was used. In addition, most of the past studies were not conducted in real communities, as past studies were conducted in external places such as in a training center [7, 8]. But the present study was conducted in real settings, which increased the ability to address real community determinants in their daily lives by improving skills among women to deal with them. A community initiative for males and females, designed to challenge gender norms and prevent violence against women and children in Uganda [37], reported that after two years following the program all forms of intimate partner violence had decreased in the community. Similarly, current research can also scale up towards a reduction of GBV incidences in a rural sector in Sri Lanka. Although evidence from evaluations in other contexts suggests that interactive group education is a key component of this type of intervention and may be necessary to sufficiently influence often deep-seated and complex gender related norms [15], the present study used more than interactive group education but also created a community driven process to convert knowledge into practice. Additionally, according to past studies economic empowerment of women was associated with risk of increasing violence. But, in our study such issues were not reported. The likely reason might be that in this intervention targeted to improve family well-being the active participation for the interventions was from women. It did not target individual level empowerment on challenging power relations but empowering women to include the family in the intervention [50, 51].

Limitations of this study include small sample size, inability to generalize findings to whole districts because of the selection of one village within it and selection of women who have a child under 5 years as the study population. Apart from the above, our study didn’t use the gender equitable men scale for gender interventions. In some similar studies they have use this scale to measure outcomes [19]. This study was too short to measure any long term changes in relation to behavior following the changes of acceptances of norms. Though, this study used only six norms, this study provides a methodology that might work with other norms as well.

Our study raises recommendations for the development of community based health promotion approaches and appropriate measures to determine their effectiveness. This study can be used as evidence for future research projects and policy measures that these kinds of initiations should be started at grassroots level health centers and grassroots level health officers should take the lead in reversing gender norms in the next generation.

## Conclusion

The findings from this study concluded that a community based health promotion intervention targeting gender norms can be used effectively to address the acceptance of gender roles among women in rural villages in Sri Lanka. These results and methodology can be applied to other communities with similar socioeconomic and demographic characteristics.

## Abbreviations

FGD: Focus Group Discussions
GBV: Gender Based Violence
IAQ: Interviewer Administered Questionnaire
LFW: Logical Frame Work
LKR: Sri Lankan Rupees
LMIC: Low and Middle Income Countries
MOH: Medical Officer of Health
PHM: Public Health Midwife
PI: Principal Investigator
SPSS: Statistical Package for Social Sciences
UCD: Underlying Community Determinants
WHO: World Health Organization
